# Introduction to the Special Issue—Cardiothoracic Surgical Critical Care: A Future of Distinction

**DOI:** 10.3390/medicina58121781

**Published:** 2022-12-03

**Authors:** Amar M. Bhatt, Michael K. Essandoh

**Affiliations:** 1 Department of Anesthesiology, Division of Critical Care Medicine and Cardiothoracic and Vascular Anesthesia, The Ohio State University Wexner Medical Center, Columbus, OH 43210, USA; 2 Department of Anesthesiology, Division of Cardiothoracic and Vascular Anesthesia, The Ohio State University Wexner Medical Center, Columbus, OH 43210, USA

Critical care after cardiothoracic surgery is an inseparable component of any successful surgical program addressing intrathoracic pathologies, including heart failure treatment with mechanical circulatory support, and respiratory failure requiring extracorporeal membrane oxygenation (ECMO) therapy [1]. However, the importance of cardiothoracic surgical critical care is often omitted in conversations concerning history, present issues, or the future of intensive care medicine and cardiothoracic surgery. The coronavirus 2019 (COVID-19) pandemic brought cardiothoracic surgeons and intensivists to the forefront with heroic efforts implementing ECMO support and lung transplantation in the most severe cases [2,3]. The two-year marathon concluded with thousands of ECMO runs and hundreds of lung transplantations around the globe, resulting in incalculable benefits of lives saved. Moreover, innovations in care delivery, systems development, and network expansions happened almost overnight, with resulting structures serving communities for years to come [4,5,6]. The COVID-19 pandemic illustrated the unique expertise and importance of cardiothoracic surgical critical care for the local and nationwide communities.

Although successful, more work remains to be done to grow and expand the field of cardiothoracic surgical intensive care. It is our belief that the care provided in cardiothoracic surgical intensive care units (CT-ICUs) is unique and requires special considerations, including an appreciation of its history, training, current knowledge gaps, and future goals. In this Special Issue of Medicina, first, the history and evolution of critical care after cardiac surgery is described. Second, readers are invited to concentrate on the specific training and education required to provide patients with the best cardiothoracic critical care. Further analysis concentrates on the appraisal of current knowledge gaps in the field, followed by future goals and strategies. Finally, the issue concludes with a review of trailblazing therapy for out-of-hospital cardiac arrest, with extracorporeal cardiopulmonary resuscitation (E-CPR). We hope you have a stimulating read!
